# Intracranial pressure based decision making: Prediction of suspected increased intracranial pressure with machine learning

**DOI:** 10.1371/journal.pone.0240845

**Published:** 2020-10-21

**Authors:** Tadashi Miyagawa, Minami Sasaki, Akira Yamaura

**Affiliations:** 1 Department of Pediatric Neurosurgery, Matsudo City General Hospital, Matsudo, Japan; 2 Department of Neurosurgery, Matsudo City General Hospital, Matsudo, Japan; University of Melbourne, AUSTRALIA

## Abstract

**Background:**

Repeated invasive intracranial pressure (ICP) monitoring is desirable because many neurosurgical pathologies are associated with elevated ICP. On the other hand, it could become a risk for children to repeat sedation, anesthesia, or radiation exposure. As a non-invasive method, measurements of optic nerve sheath diameter (ONSD) has been revealed to accurately predict increased ICP. However, no studies have indicated a relationship among age, brain, and ventricular parameters in normal children, nor a prediction of increased ICP with artificial intelligence.

**Methods and findings:**

This study enrolled 400 normal children for control and 75 children with signs of increased ICP between 2009 and 2019. Measurements of the parameters including ONSD on CT were obtained. A supervised machine learning was applied to predict suspected increased ICP based on CT measurements. A linear correlation was shown between ln(age) and mean ONSD (mONSD) in normal children, revealing mONSD = 0.36ln(age)+2.26 (R^2^ = 0.60). This study revealed a linear correlation of mONSD measured on CT with ln(age) and the width of the brain, not the width of the ventricles in 400 normal children based on the univariate analyses. Additionally, the multivariate analyses revealed minimum bicaudate nuclei distance was also associated with mONSD. The results of the group comparison between control and suspected increased ICP revealed a statistical significance in mONSD and the width of the ventricles. The study indicated that supervised machine learning application could be applied to predict suspected increased ICP in children, with an accuracy of 94% for training, 91% for test.

**Conclusions:**

This study clarified three issues regarding ONSD and ICP. Mean ONSD measured on CT was correlated with ln(age) and the width of the brain, not the width of the ventricles in 400 normal children based on the univariate analyses. The multivariate analyses revealed minimum bicaudate nuclei distance was also associated with mONSD. Mean ONSD and the width of ventricles were statistically significant in children with signs of elevated ICP. Finally, the study showed that machine learning could be used to predict children with suspected increased ICP.

## Introduction

In the clinical settings of pediatric neurosurgery, many pathologies are associated with increased intracranial pressure (ICP). For the initial diagnosis and follow-up evaluations of many underlying neurosurgical conditions, repeated invasive ICP measurements are desirable; however, it could become a risk for children to repeat sedation, anesthesia, or radiation exposure. Therefore, a reliable and useful clinical technique to non-invasively assess the ICP status in children is admirable [1, 2].

Recent studies have indicated the efficacy of various non-invasive methods for predicting elevated ICP [1–5]. In their study, measurements of optic nerve sheath diameter (ONSD) with ultrasound, CT, or MRI could accurately predict increased ICP. The optic nerve sheath is anatomically continuous with intracranial dura mater, with trabeculated subarachnoid space in which cerebrospinal fluid exists [6]. Therefore, changes in ICP are transmitted directly to the space around the optic nerve. As ICP increases, CSF moves to the expandable optic nerve sheath and optic nerve sheath expands [7, 8]. ONSD measurements have been revealing to correlate with ICP in children [9, 10]. However, no studies have indicated a relationship among age, brain, and ventricular parameters in normal children.

Artificial intelligence (AI) uses computer systems to simulate cognitive abilities to achieve goals. Machine learning classification is one of the domains of AI that enables an algorithm or classifier to learn patterns in large, complex datasets and produce useful predictive outputs. The number of published machine learning studies in neurosurgery is increasing exponentially [11–14]. Some of them have focused on the application of machine learning algorithms to support clinical decision making in neurosurgery. However, no studies have yet been published regarding the use of machine learning to predict increased ICP.

The purpose of this study was to identify three issues regarding ONSD and suspected elevated ICP. First, to determine the relationship between ONSD and age, the brain width, or the ventricular width by CT in normal children. Secondly, we performed an analysis to detect differences between normal children and children with clinical signs of elevated ICP. Finally, the feasibility of machine learning to predict children with suspected elevated ICP was examined.

## Materials and methods

This is a retrospective study of a prospectively collected database on children visited or admitted to our hospital between 2009 and 2019. The inclusion criteria are as follows; the control group (CTR) included 400 children who visited our hospital because of minor head injury or other minor events, revealed no intracranial or skull abnormal lesion on CT of the head. The exclusion criteria included children with previously diagnosed intracranial lesions. The suspected increased intracranial pressure (siICP) group enrolled 75 patients who presented with signs of elevated ICP, such as disturbance of consciousness, confusion, irritability, increased sleepiness, shrill or high-pitched cry, headache, nausea/vomiting, bulging of anterior fontanelle, and convulsion. A board-certified pediatric neurosurgeon (TM) and board-certified neurosurgeons confirmed children with at least 2 signs of increased ICP as an siICP.

### Measurement protocol on CT

**Fig 1:**
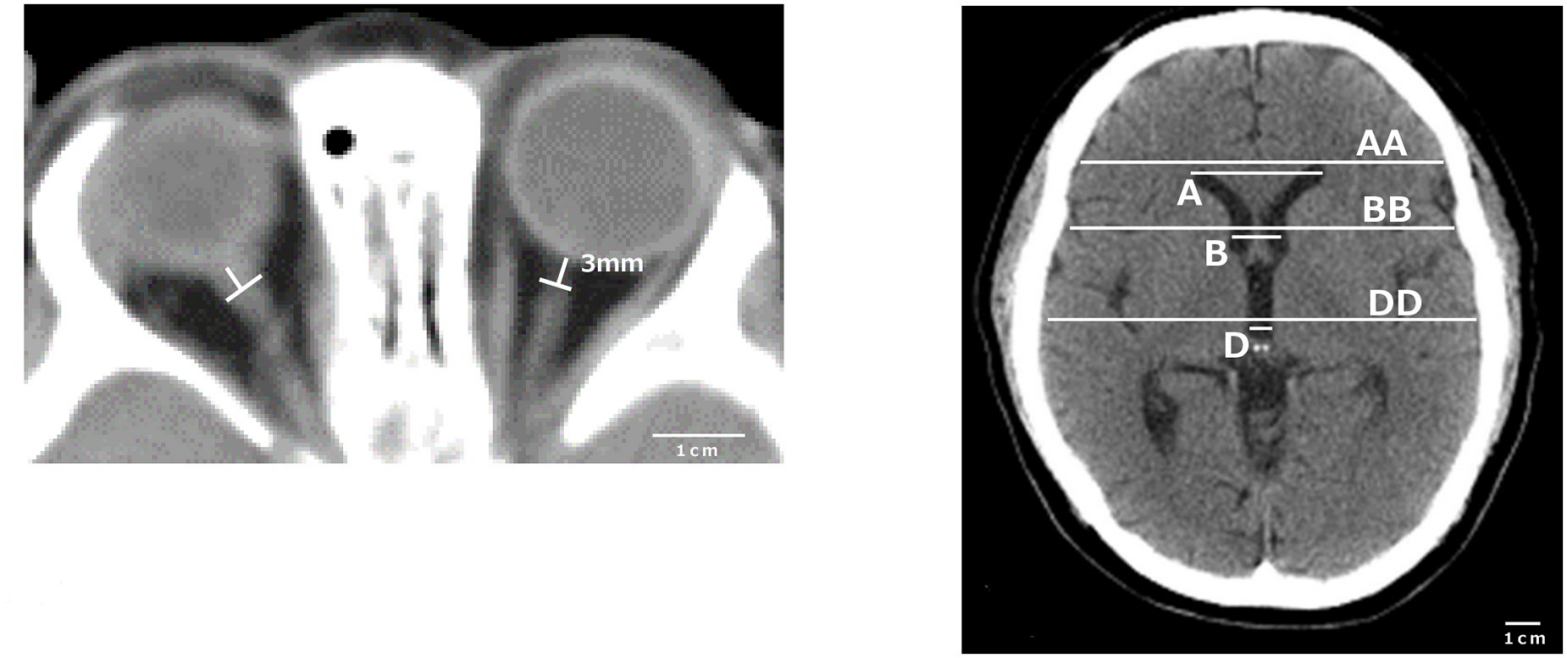
Computed Tomography imaging. The optic nerve sheath diameter was measured 3 mm behind the posterior aspect of the globe on axial sequences. A: Maximum distance between anterior horn, B: Minimum bicaudate nuclei distance, D: Third ventricle distance. AA, BB and DD were corresponding brain width, respectively. EE defined a square root of summation for AA2, BB2, and DD2.

Measurements of parameters, including ONSD, were performed under a rigorous protocol established by our team to minimize interobserver variability. Our protocol includes several points: using the same application software on our PACS system, selecting CT images with maximum optic nerve visibility, using the same magnification and the same density, and measuring three times to minimize interobserver variability. The ONSD was measured 3 mm behind the posterior aspect of the globe on axial sequences (Fig 1) using the fat window for ONSD and brain window for other parameters. The actual measurement using the software was achieved to measure from the gray-black border to the opposite gray-black border of the optic nerve (gray; optic nerve sheath, black; intraorbital fat). Bilateral ONSD was measured in all patients, then calculated mean ONSD which was defined as the average value of the left and right ONSD. The other 6 parameters included the width of the brain and the ventricles in each level were measured as shown in Fig 1. (A: maximum distance between anterior horn, B: minimum bicaudate nuclei distance, D: third ventricle distance, AA, BB, and DD: corresponding brain width, respectively, EE: the square root of summation for AA^2^, BB^2^, and DD^2^, *ie* $E=\sqrt{{AA}^{2}+{BB}^{2}+{DD}^{2}}$).

### Data analysis

For univariate analysis, Fisher’s exact test was used to determine significance, which was defined as p < 0.05. This study was satisfied when the statistical power is 0.8 or higher, corresponding to an 80% chance of detecting a real effect of the expected size. Also, multiple regression analysis and Spearman’s rank correlation analysis were applied to investigate the relationship between CT parameters.

### Machine learning

A supervised machine learning in which the program was written by python was applied to predict suspected increased ICP based on ln(age) and the measurements on CT. The logistic analytical method was used in the algorithm to classify the children into intact and suspected elevated ICP. In detail, 80 percent of the total 475 cases were used for training, 20% for test. A combination of the values among ln(age), mONSD, A, B, D, AA, BB, DD, or EE was used for maximum accuracy with as few values as possible. The precision of the algorithm was evaluated by calculating accuracy.

This study complies with the standards of the Declaration of Helsinki and the current ethical guidelines. The study also was approved by the institutional ethics board and by the IRB of Matsudo City General Hospital. Verbal consent was obtained from the caregivers for using the data.

## Results

Table 1 showed the demographic features in the control and the siICP group. The mean age of all cases and females in siICP was older than that in the control group, no statistical significance, however, was observed.

**Table 1:** Demographic features in control and suspected increased intracranial pressure group.

	Control	siICP
Total number	400	75
male	258	53
female	142	22
Age (days)	844.7±1473.4	1080.4±1781.9
male	771.7±1278.6	774.6±1374.8
female	977.3±1770.9	1817.2±2389.7

**Fig 2:**
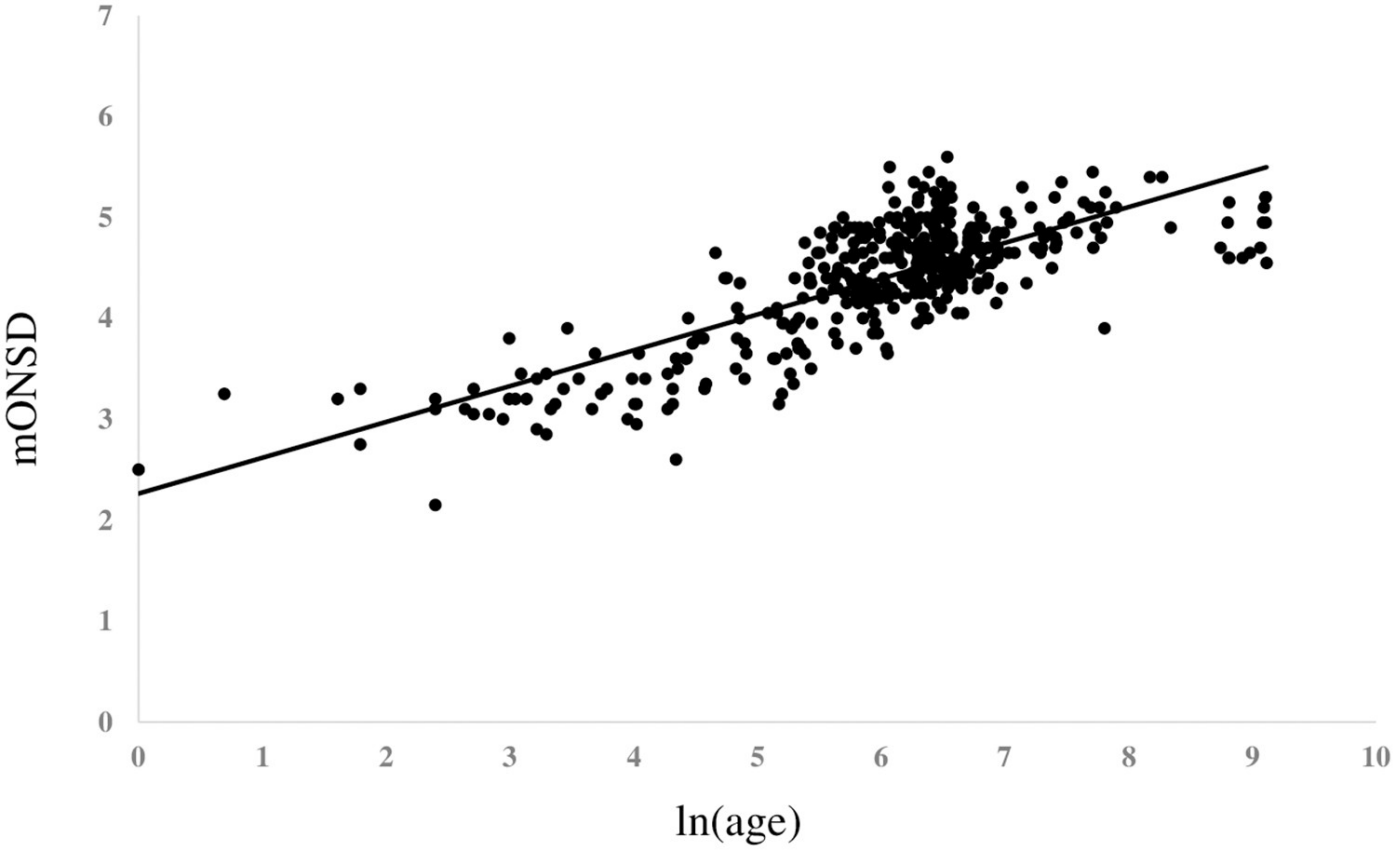
Optic nerve sheath diameter of those in normal control plotted in relation to ln(age). Results of regression analysis and Spearman’s rank correlation analysis is shown in separate table. mONSD; mean optic nerve diameter, ρ; Spearman’s rank correlation coefficient.

**Fig 3:**
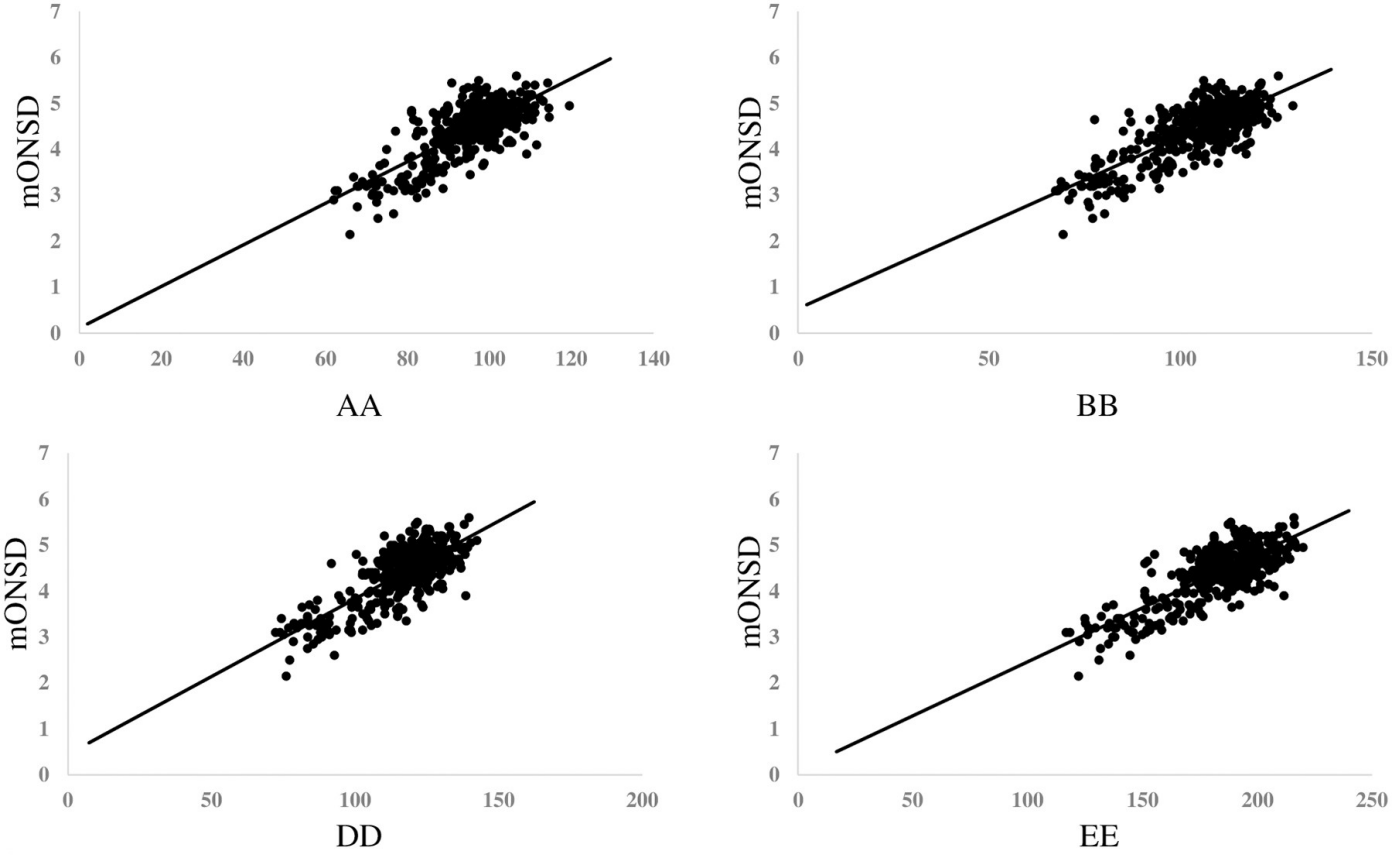
Mean optic nerve sheath diameter of those in normal control plotted in relation to width of the brain, revealing that mONSD was linearly correlated with AA-EE, respectively. Results of regression analysis and Spearman’s rank correlation analysis is shown in separate table. mONSD; mean optic nerve sheath diameter, AA, BB and DD; corresponding brain width to maximum distance between anterior horn, minimum bicaudate nuclei distance, third ventricle distance, respectively, EE: a square root of summation for AA2, BB2, and DD2, ρ; Spearman’s rank correlation coefficient.

**Fig 4:**
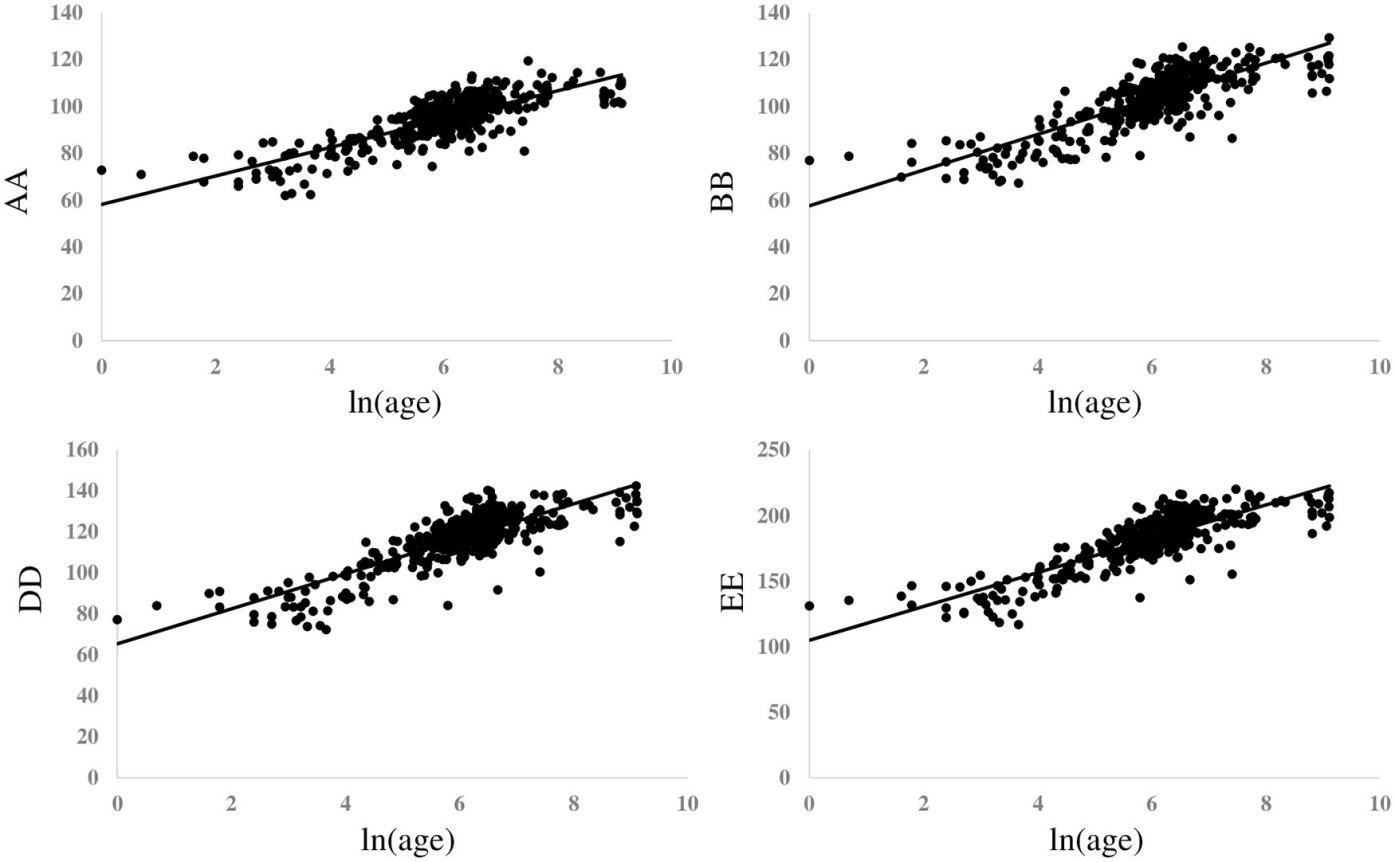
Width of the brain of those in normal control plotted in relation to ln(age), revealing that AA, BB, DD and EE were linearly correlated with ln(age), respectively. Results of regression analysis and Spearman’s rank correlation analysis is shown in separate table. AA, BB and DD: corresponding brain width to maximum distance between anterior horn, minimum bicaudate nuclei distance, third ventricle distance, respectively, EE: a square root of summation for AA2, BB2, and DD2, ρ; Spearman’s rank correlation coefficient.

**Fig 5:**
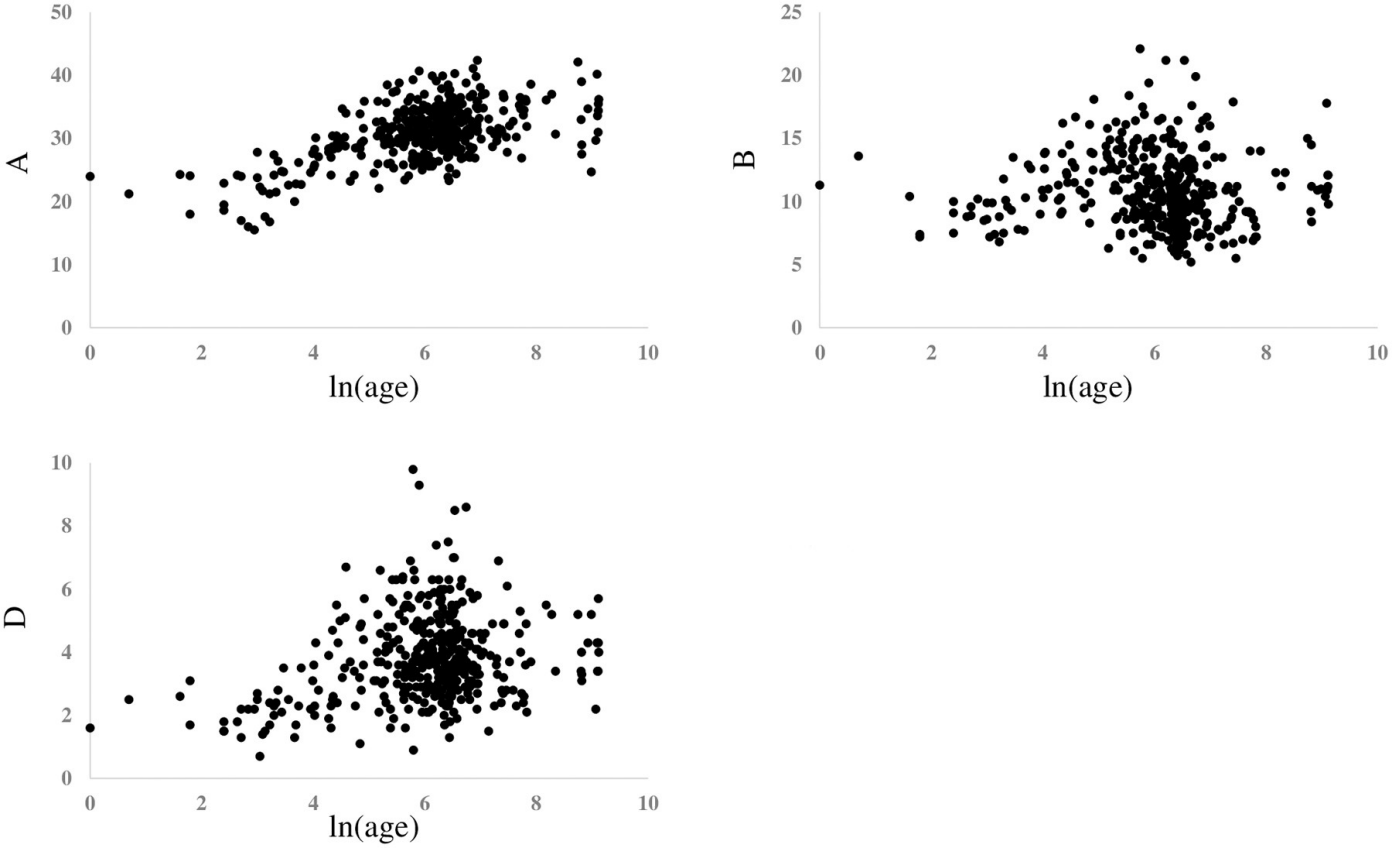
Width of the ventricle of those in normal control plotted in relation to ln(age), revealing that A was weakly correlated with ln(age), however absent in linear correlation in B and D. Results of regression analysis and Spearman’s rank correlation analysis is shown in separate table. A: Maximum distance between anterior horn, B: Minimum bicaudate nuclei distance, D: Third ventricle distance.

**Fig 6:**
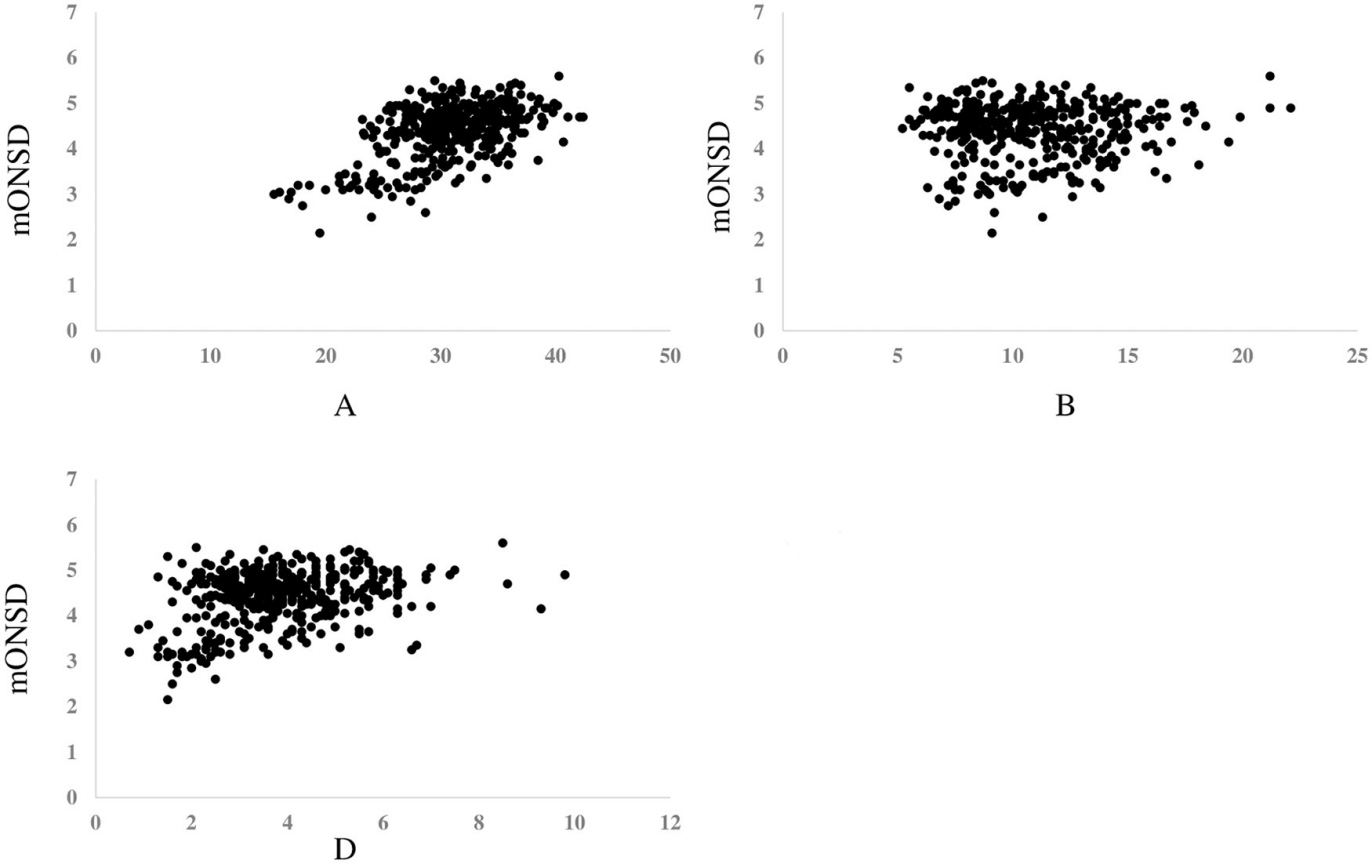
Mean optic nerve sheath diameter of those in normal control plotted in relation to the width of the ventricles, revealing that mONSD was weakly correlated with A, however absent in linear correlation in B and D. Results of regression analysis and Spearman’s rank correlation analysis is shown in separate table. mONSD; mean optic nerve sheath diameter, A: Maximum distance between anterior horn, B: Minimum bicaudate nuclei distance, D: Third ventricle distance.

**Fig 7:**
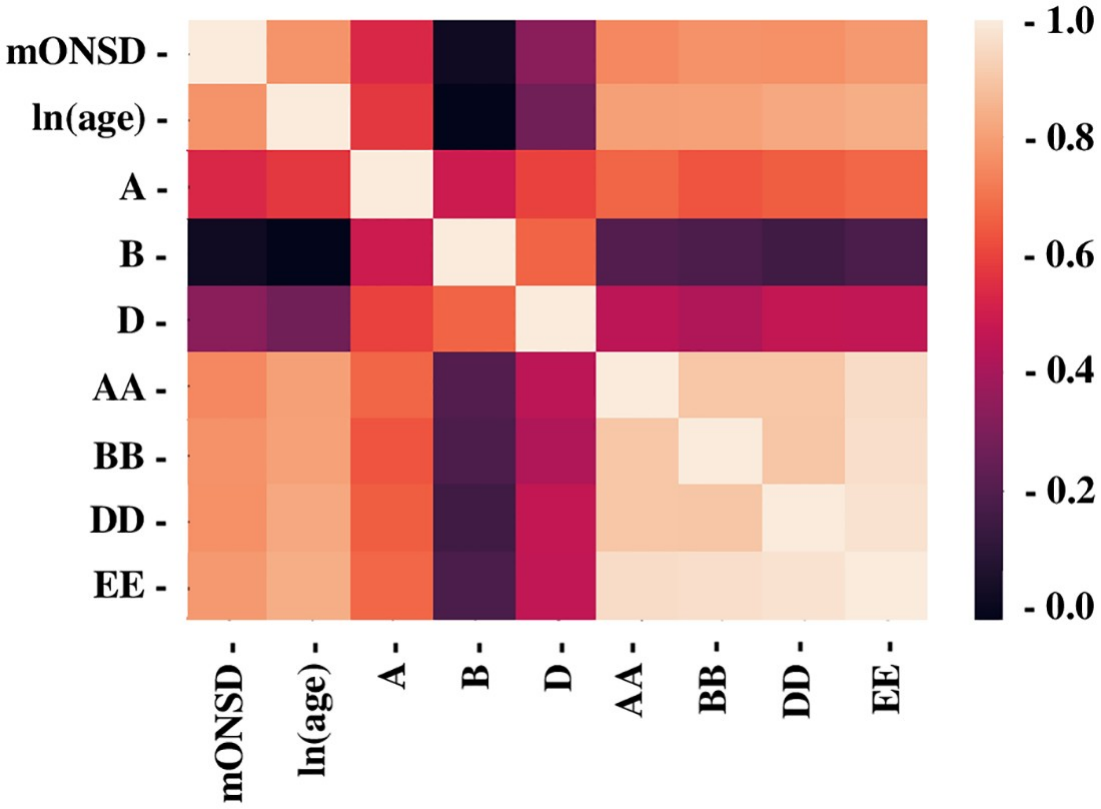
Heatmap of the multivariate analysis results. The correlation coefficient was expressed in heatmap fashion. Lower coefficient was shown in darker, higher in lighter. mONSD; mean optic nerve sheath diameter, A: Maximum distance between anterior horn, B: Minimum bicaudate nuclei distance, D: Third ventricle distance. AA, BB and DD: corresponding brain width to A, B and D, respectively, EE: a square root of summation for AA^2^, BB^2^, and DD^2^.

After converting the day of life to the logarithmic expression, ie ln(age), Fig 2 showed the graph revealing the relationship between ln(age) and mONSD in 400 normal children. The range was 2.15–5.60 mm with a mean of 4.39 (SD 0.61) mm. An analysis revealed the correlation between increasing ln(age) and increasing mONSD. It also indicated mONSD = 0.36ln(age)+2.26 (R2 = 0.60). Spearman’s rank correlation analysis showed 0.67 in the correlation coefficient (p < 0.01). In terms of mONSD of those in normal control plotted to the width of the brain, Fig 3 demonstrated that mONSD was linearly correlated with AA-EE, respectively. Additionally, the width of the brain was a function of ln(age) as shown in Fig 4. However, the absence of a linear relationship or only a weak linear correlation was observed in A, B, and D when plotting the width of the ventricles to ln(age) (Fig 5). Fig 6 also demonstrated that mONSD has no linear relationship or only a weak correlation with the width of the ventricles. Summarizing the univariate analysis, mONSD was a function of age and also the width of the brain, but not the width of the ventricles. Fig 7 and Table 2 showed the multivariate analytic data. Fig 7 demonstrated a correlation coefficient heatmap regarding ln(age) and the CT parameters. This also showed a strong relation between mONSD and AA, BB, DD, or EE. Multiple regression analysis in the control group revealed no difference between 2 scenarios as shown in Table 2A and 2B (scenario1; explanatory variables included ln(age), A, B, D, AA, BB and DD, scenario 2; explanatory variables included ln(age), A, B, D, and EE). Therefore, scenario 2 was used in further analysis because of fewer explanatory variables. Then, mONSD = 0.153ln(age) + 0.006A − 0.029B + 0.041D + 0.014EE with R2 = 0.67 and significant F value < 0.01. The analysis also revealed the correlation coefficient of ln(age), B, and EE were significant at the level of p < 0.01. Table 2C showed a bit lower coefficient of determination in females compared to that in males.

**Table 2:** Results of multiple regression analysis, revealing no significant difference between scenario1 and 2. Therefore, decision was made to use scenario 2 in the further analysis because of fewer explanatory variables. Scenario 1; explanatory variables include ln(age), A, B, D, AA, BB and DD, Scenario 2; explanatory variables include ln(age), A, B, D and EE, n; case number, R; correlation coefficient, R^2^; coefficient of determination, SE; standard error, A: Maximum distance between anterior horn, B: Minimum bicaudate nuclei distance, D: Third ventricle distance, AA, BB and DD: corresponding brain width to maximum distance between anterior horn, minimum bicaudate nuclei distance, third ventricle distance, respectively, EE: a square root of summation for AA^2^, BB^2^, and DD^2^.

**(A)**								
	scenario 1				scenario 2			
N	400				400			
multiple regression R	0.82				0.82			
multiple regression R^2^	0.68				0.67			
SE	0.35				0.35			
significant F value	<0.01				<0.01			
**(B)**								
	scenario 1				scenario 2			
	coefficient	SE	t	p value	coefficient	SE	t	p value
intercept	1.027	0.21	4.96	<0.01	0.935	0.20	4.67	<0.01
ln(age)	0.153	0.03	5.79	<0.01	0.153	0.03	5.78	<0.01
A	0.007	0.01	1.14	0.25	0.006	0.01	0.97	0.33
B	0.031	0.01	3.42	<0.01	0.029	0.01	3.21	<0.01
D	0.047	0.02	2.36	0.02	0.041	0.02	2.10	0.04
AA	0.002	0.00	0.45	0.65				
BB	0.015	0.00	3.92	<0.01				
DD	0.006	0.00	1.55	0.12				
EE					0.014	0.00	7.56	<0.01
**(C)**								
	Total				Male		Female	
N	400				258		142	
multiple regression R	0.82				0.83		0.80	
multiple regression R^2^	0.67				0.69		0.64	
SE	0.35				0.35		0.34	
significant F value	<0.01				<0.01		<0.01	

Table 3 showed the results of the group comparison between the control and the siICP. It revealed statistical significance in mONSD, A, B, and D between the control and the siICP.

**Table 3:** Group comparison between control and suspected increased intracranial pressure.

	Control	siICP	p
ln(age)	6.0±1.3	5.7±2.0	0.08
mONSD(mm)	4.4±0.6	5.3±1.1	<0.01
AA(mm)	94.6±10.0	93.9±13.4	0.60
BB(mm)	103.3±12.5	101.6±16.4	0.31
DD(mm)	116.6±13.8	116.0±17.1	0.75
EE(mm)	182.3±20.4	180.7±26.2	0.55
A(mm)	30.9±4.5	33.9±8.3	<0.01
B(mm)	10.8±3.0	15.2±7.5	<0.01
D(mm)	3.8±1.4	6.1±4.2	<0.01

**Fig 8:**
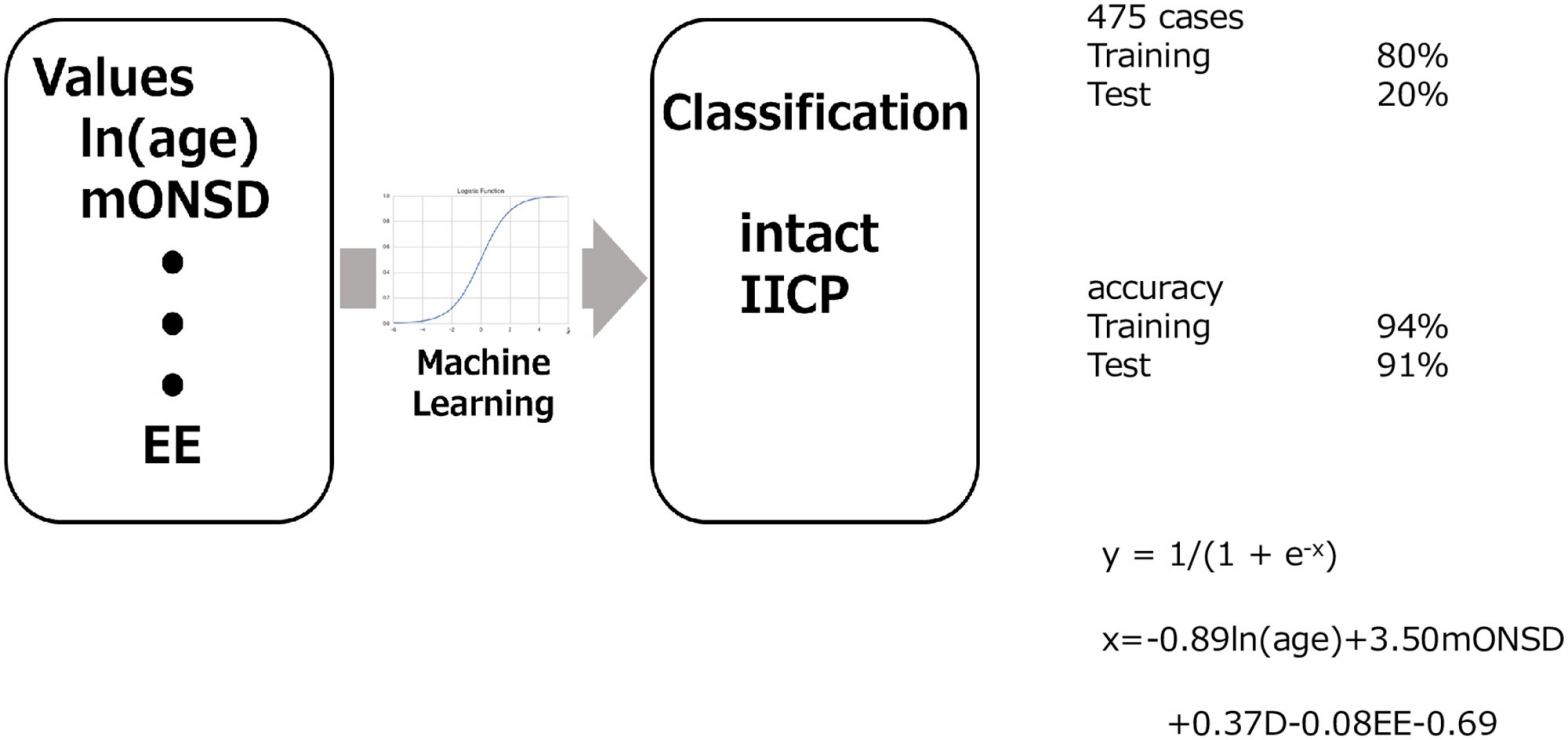
Schematic figure of supervised machine learning and the results, showing accuracy of machine learning application was 94% for training, and 91% for test. Also it shows the logistic equation obtained, y = 1/(1+e^-x^), x = -0.89ln(age)+3.50mONSD+0.37D-0.08EE-0.69.

Supervised machine learning was applied to classify the children to predict suspected raised ICP as shown in Fig 8. A code was written in python using the logistic analytical method to classify them into 2 class, intact and siICP. The values of ln(age), mONSD, A, B, D, AA, BB, DD, and EE were combined to obtain maximum accuracy with as few values as possible. Eighty percent of 475 cases were used for training, 20% for test. The analysis revealed only 4 values, ln(age), mONSD, D, and EE were applied to make the accuracy of 94% for training, 91% for test. The logistic equation obtained was x = -0.89ln(age) + 3.50mONSD + 0.37D − 0.08EE -0.69, probability y = 1/(1+e^-x^).

## Discussion

This study identified three issues regarding ONSD and suspected elevated ICP. First of all, the study revealed a relationship among ONSD measured on CT and ln(age), parameters of the brain, or the ventricles in 400 normal children. Secondly, measurement of the parameters including ONSD on CT showed that ONSD and the width of ventricles were statistically significant in children with signs of elevated ICP compared to control children. Finally, the study showed that machine learning could be used to predict children with suspected increased ICP.

### Relationship of parameters measured on CT in control children

Fig 2 shows the relationship between ln(age) and mONSD in control children. The correlation analysis indicated mONSD = 0.36ln(age)+2.26 (R2 = 0.60). In the previous studies [15–17] using ultrasound or MR imaging, logarithmic regression analysis demonstrated a relationship between increasing age and increasing ONSD, although they enrolled quite a few infants and younger children. This study using CT of the head in 400 normal children demonstrated a linear correlation between ln(age) and mONSD. The mONSD in the control group was also linearly correlated with the width of the brain, not the width of the ventricles. As shown in Fig 4, the width of the brain is a function of ln(age). Taken together with the results in Figs 2, 3 and 4, mONSD is a function of ln(age) because there is a correlation between mONSD and the brain width (Fig 3), and the brain width and ln(age) (Fig 4), respectively. The analysis also revealed that mONSD did not correlate linearly with the width of the ventricles in normal children, and the results of the univariate analysis suggest that mONSD appears to be age-dependent in normal children, rather than the morphology of the ventricles or the volume of the cerebrospinal fluid space.

The results of the multivariate analysis revealed the relationship between each parameter, as shown in Fig 7. The heatmap for the correlation coefficient confirmed the results of univariate analysis, indicating the strong relationship between mONSD and AA, BB, DD, or EE. When considering 2 scenarios (scenario1; explanatory variables included ln(age), A, B, D, AA, BB and DD, scenario 2; explanatory variables included ln(age), A, B, D, and EE), multiple regression analysis in the control group showed no significant difference between 2 scenarios. This result indicated that EE instead of AA, BB, and DD could be used in further analyses with the same statistical values of R^2^ and F value. The multiple regression equation obtained could explain 67% of mONSD. The analysis also revealed the correlation coefficient of ln(age), B, and EE were significant at the level of p < 0.01, indicating those 3 elements significantly contribute to the equation. The mONSD in the control children could be speculated mathematically with age, minimum bicaudate nuclei distance, and the square root of 3 brain widths. Based on the univariate and multivariate analyses, mONSD was speculated to correlate strongly to ln(age) and the brain width, and fairly to the ventricular width. To the best of our knowledge, this is the first report which revealed a relationship between ONSD and the CT parameters including the width of the brain or the ventricles.

Although a slightly lower coefficient of determination was observed in females than in males (Table 2C), the F value was still significant. Further studies, including more female cases, would be needed in the future.

### Group comparison between the control and the siICP

As shown in Table 3, there are significant differences between the control and the siICP regarding mONSD and the width of the ventricles. This suggests that any intracranial lesions inducing the ICP elevation have, to some degree, ventricular dilatation, which in turn causes the shift of CSF toward extracranial CSF space such as the subarachnoid space around the optic nerve. An enlarged ONSD, as a reflection of ICP, is a concept that has been evolving throughout the past 25 years. The rationale behind this concept is derived from the anatomic extension of the subarachnoid space underneath the optic nerve sheath and its connection to the CSF cavities. Increased ICP is, therefore, believed to cause transmission of force through this space, resulting in distension of the ONSD [6–8].

Several studies have found a correlation between ONSD on ultrasound [1, 2, 16, 17], MRI [15], CT [18], and ICP measured through traditional monitoring. In the previous studies which enrolled adult patients with any kind of intracranial pathologies, Robba et al. showed that ONSD measured on ultrasound had the strongest correlation in predicting increased ICPs in patients with invasive ICP monitoring [19]. Kimberly and the colleagues revealed in their study that an ONSD > 5.0 mm had a sensitivity and specificity of 88% and 93% in detecting ICP > 20 cmH2O in patients with an ICP monitor [20]. Additionally, Raffiz and Abdullah indicated that the ONSD cutoff of 5.2 mm had a 95% sensitivity and an 80% specificity in predicting increased ICPs [21]. Kerscher and her colleagues showed the relation of ONSD and ICP in pediatric neurosurgery practice [1, 2]. They indicated that children > 1 year showed a better correlation between ONSD and ICP, and those < 1 year did worse while infants with open fontanelle had no correlation. Although our study used clinical signs, such as headache, nausea/vomit, bulging anterior fontanelle, etc. as indicators for suspected elevated ICP, the results showed a significance between the control and the siICP. In the previous study conducted by Siddiqui et al. [22], there had a relation with good sensitivity and specificity between clinical signs of the raised ICP and ultrasonic ONSD measurements.

### Prediction of suspected increased ICP with supervised machine learning

Based on the results mentioned above, we tried to use supervised machine learning to classify the children into 2 groups, intact and siICP, using ln(age) and CT parameters.

The values of ln(age), mONSD, A, B, D, AA, BB, DD, and EE were combined to obtain maximum accuracy with as few values as possible. Among these values, the analysis revealed only 4 values, ln(age), mONSD, D, and EE, were the ones for obtaining the maximum accuracy. It revealed an accuracy of 94% for training, 91% for test. The logistic equation obtained was x = -0.89ln(age) + 3.50mONSD + 0.37D − 0.08EE—0.69, probability y = 1/(1+e^-x^). To the best of our knowledge, our study presents the first to predict suspected increased ICP in children using an AI, especially supervised machine learning. When inserting ln(age), mONSD, D, and EE obtained from CT of the head in the child whom we would like to know whether he or she may have suspected increased ICP into the equation, it automatically reveals a probability of suspected elevated ICP with high accuracy. This study indicated that the supervised machine learning could be used to predict suspected increased ICP in children. Although machine learning-based systems are powerful technologies as mentioned above, they should not replace the clinical judgment of physicians and medical teams [11–14]. The ideal role of these systems is as a data-driven input to the surgical decision-making process, designed to solve focused problems such as predicting the risk of increased ICP in this study. The question to consider in neurosurgery is what would be when a neurosurgeon trusts on the guidance of a machine learning-driven clinical decision support system and the patient suffers an unexpectedly poor outcome. A support system regarding clinical decisions must only be used by professionals and should be just one of many inputs to the decision-making process. To maximize safety, machine learning models should be developed according to a robust process [11].

### Limitation of this study

In terms of demographic characteristics, the number of female children was relatively small compared to the number of male children. Therefore, this issue may affect the average age (Table 1) or the interpretation of the results of this study. Better age management would be needed in future studies, especially for females. Validation was not included in this study and future studies will need to be prospective. Finally, studies in the future will clarify a relationship between mONSD and invasively measured ICP. However, because the present algorithm included elements of age and mONSD, machine learning was accurately able to predict the suspected elevated ICP.

Although ultrasound has been used to measure ONSD, its advantages include ease of use at the bedside and in the emergency room and no radiation exposure. On the other hand, a disadvantage is that there are the interobserver and intraobserver errors. Because the main focus of this study was to reveal the relationship between ONSD and the brain, the head CT was used. However, there is the possibility of performing an ultrasound technique with standardized A-scan technology and a "30 degree test" that can be used to obtain more accurate measurements of the optic nerve sheath [23]. The ONSD measured using this technique will be compared to the ONSD in our study in the future.

## Conclusion

This study clarified three issues regarding mONSD and suspected elevated ICP. First of all, this study revealed a linear correlation of mONSD measured on CT with ln(age) and the width of the brain, not the width of the ventricles in 400 normal children based on the univariate analyses. Additionally, the multivariate analyses revealed minimum bicaudate nuclei distance was also associated with mONSD. Second of all, measurement of the parameters including ONSD on CT showed that ONSD and the width of the ventricles were statistically significant in children with signs of elevated ICP as compared with control children. Finally, the study showed that machine learning could be used to predict children with suspected increased ICP.

## Supporting information

S1 Table (TIF)

S2 Table (TIF)

S3 Table (TIF)

S4 Table (TIF)

S5 Table (TIF)

## References

[1] Kerscher SR, Schöni D, Hurth H, Neunhoeffer F, Haas-Lude K, Wolff M, et al The relation of optic nerve sheath diameter (ONSD) and intracranial pressure (ICP) in pediatric neurosurgery practice—Part I: Correlations, age-dependency and cut-off values. Childs Nerv Syst. 2020;36(1):99–106. 10.1007/s00381-019-04266-1 31256241

[2] Kerscher, Schöni D, Neunhoeffer F, Wolff M, Haas-Lude K, Bevot A, et al The relation of optic nerve sheath diameter (ONSD) and intracranial pressure (ICP) in pediatric neurosurgery practice—Part II: Influence of wakefulness, method of ICP measurement, intra-individual ONSD-ICP correlation and changes after therapy. Childs Nerv Syst. 2020;36(1):107–115. 31392457 10.1007/s00381-019-04336-4

[3] Lee HC, Lee WJ, Dho YS, Cho WS, Kim YH, Park HP, et al Optic nerve sheath diameter based on preoperative brain computed tomography and intracranial pressure are positively correlated in adults with hydrocephalus. Clin Neurol Neurosurg. 2018;167:31–5. 10.1016/j.clineuro.2018.02.012 29433056

[4] Maissan IM, Dirven PJ, Haitsma IK, Hoeks SE, Gommers D, Stolker RJ. Ultrasonographic measured optic nerve sheath diameter as an accurate and quick monitor for changes in intracranial pressure. J Neurosurg. 2015;123:743–7. 10.3171/2014.10.JNS141197 25955869

[5] Mehrpour M, Torshizi FO, Esmaeeli S, Taghipour S, Abdollahi S. Optic nerve sonography in the diagnostic evaluation of pseudopapilledema and raised intracranial pressure: A cross-sectional study. Neurol Res Int.2015;146059 10.1155/2015/146059 25874128 PMC4385686

[6] Killer HE, Jaggi GP, Flammer J, Miller NR, Huber AR, Mironov A. Cerebrospinal fluid dynamics between the intracranial and the subarachnoid space of the optic nerve. Is it always bidirectional? Brain. 2007;130(Pt 2):514–20. 10.1093/brain/awl324 17114796

[7] Hansen HC, Helmke K. The subarachnoid space surrounding the optic nerves. An ultrasound study of the optic nerve sheath. Surg Radiol Anat. 1996;18(4):323–8. 10.1007/BF01627611 8983112

[8] Helmke K, Hansen HC. Fundamentals of transorbital sonographic evaluation of optic nerve sheath expansion under intracranial hypertension. I. Experimental study. Pediatr Radiol. 1996;26(10):701–5. 10.1007/BF01383383 8805599

[9] Padayachy LC, Padayachy V, Galal U, Gray R, Fieggen AG. The relationship between transorbital ultrasound measurement of the optic nerve sheath diameter (ONSD) and invasively measured ICP in children: Part I: repeatability, observer variability and general analysis. Childs Nerv Syst. 2016;32(10):1769–78. 10.1007/s00381-016-3067-5 27659819

[10] Padayachy LC, Padayachy V, Galal U, Pollock T, Fieggen AG. The relationship between transorbital ultrasound measurement of the optic nerve sheath diameter (ONSD) and invasively measured ICP in children. Part II: age-related ONSD cut-off values and patency of the anterior fontanelle. Childs Nerv Syst. 2016;32(10):1779–85. 10.1007/s00381-016-3068-4 27659820

[11] Buchlak QD, Esmaili N, Leveque JC, Farrokhi F, Bennett C, Piccardi M, et al Machine learning applications to clinical decision support in neurosurgery: an artificial intelligence augmented systematic review. Neurosurgical Review. 2019; 17. 10.1007/s10143-019-01163-8 31422572

[12] Senders JT, Arnaout O, Karhade AV, Dasenbrock HH, Gormley WB, Broekman ML, et al Natural and Artificial Intelligence in Neurosurgery: A Systematic Review. Neurosurgery. 2018;83(2):181–192. 10.1093/neuros/nyx384 28945910

[13] Senders JT, Zaki MM, Karhade AV, Chang B, Gormley WB, Broekman ML, et An introduction and overview of machine learning in neurosurgical care. Acta Neurochir (Wien). 2018;160(1):29–38. 29134342 10.1007/s00701-017-3385-8

[14] Celtikci E. A Systematic Review on Machine Learning in Neurosurgery: The Future of Decision-Making in Patient Care. Turk Neurosurg. 2018;28(2):167–173. 10.5137/1019-5149.JTN.20059-17.1 28481395

[15] Shofty B, Ben-Sira L, Constantini S, Freedman S, Kesler A. Optic Nerve Sheath Diameter on MR Imaging: Establishment of Norms and Comparison of Pediatric Patients with Idiopathic Intracranial Hypertension with Healthy Controls. AJNR 2012; 33(2): 366–369. 10.3174/ajnr.A2779 22116116 PMC7964794

[16] Newman WD, Hollman AS, Dutton GN, Carachi R. Measurement of optic nerve sheath diameter by ultrasound: a means of detecting acute raised intracranial pressure in hydrocephalus. Br J Ophthalmol. 2002;86(10):1109–13. 10.1136/bjo.86.10.1109 12234888 PMC1771326

[17] Ballantyne J, Hollman AS, Hamilton R, Bradnam MS, Carachi R, Young DG, et al Transorbital optic nerve sheath ultrasonography in normal children. Clin Radiol. 1999;54(11):740–2. 10.1016/s0009-9260(99)91176-5 10580764

[18] Majeed G, Kashyap S, Menoni R, Miulli D, and Sweiss D. A noninvasive method for the estimation of increased intracranial pressure in patients with severe traumatic brain injury using optic nerve sheath diameter measured on computed tomography head. Surg Neurol Int. 2019; 10: 97 10.25259/SNI-120-2019 31528435 PMC6744793

[19] Robba C, Cardim D, Tajsic T, Pietersen J, Bulman M, Donnelly J, et al Ultrasound non-invasive measurement of intracranial pressure in neurointensive care: A prospective observational study. PLoS Med. 14(7):10.1371/journal.pmed.1002356 PMC5526499 28742869

[20] Kimberly HH, Shah S, Marill K, Noble V. Correlation of optic nerve sheath diameter with direct measurement of intracranial pressure. Acad Emerg Med. 2008;15(2):201–4. 10.1111/j.1553-2712.2007.00031.x 18275454

[21] Raffiz M, Abdullah JM. Optic nerve sheath diameter measurement: a means of detecting raised ICP in adult traumatic and non-traumatic neurosurgical patients. Am J Emerg Med. 2017;35(1):150–153. 10.1016/j.ajem.2016.09.044 27852525

[22] Rehman Siddiqui NU, Haque A, Abbas Q, Jurair H, Salam B, Sayani R. Ultrasonographic optic nerve sheath diameter Measurement for raised intracranial pressure in a Tertiary care centre of a developing country. J Ayub Med Coll Abbottabad. 2018; 30(4):495–500. 30632323

[23] Vitiell L, Bernardo M, Nuzio SG, Mandato C, Rosa N, Vajro P. Pediatric liver diseases and ocular changes: What hepatologists and ophthalmologists should know and share with each other. Dig Liver Dis. 2020;52(1):1–8. 10.1016/j.dld.2019.11.009 31843253

