# Supplementary figures and images for: Intracranial pressure based decision making: Prediction of suspected increased intracranial pressure with machine learning
Source: PLoS One. 2020 Oct 21;15(10):e0240845. doi: 10.1371/journal.pone.0240845 (PMC7577462; doi:10.1371/journal.pone.0240845)

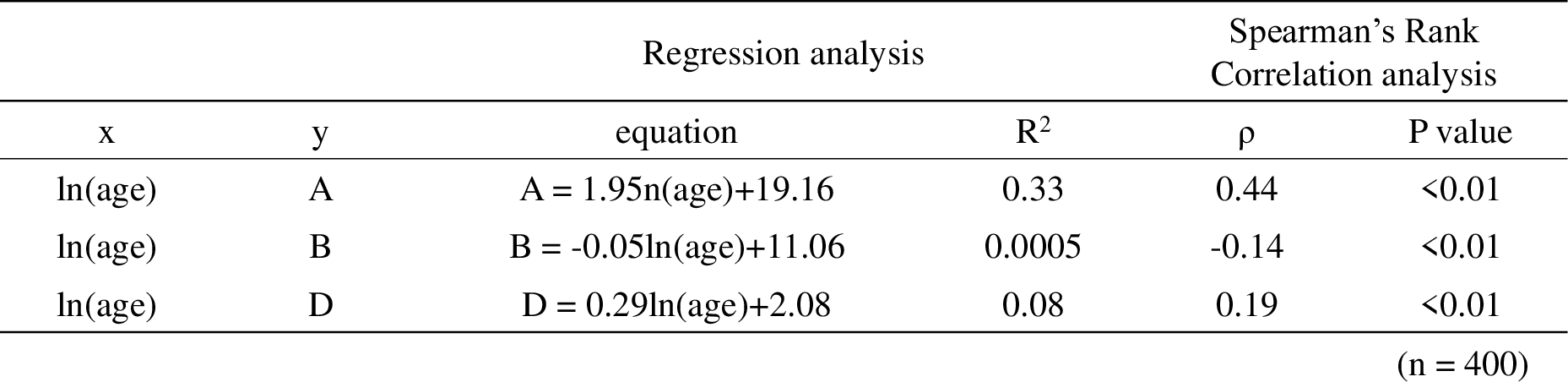

Supplement: S1 Table — (TIF) [file pone.0240845.s001.tif]

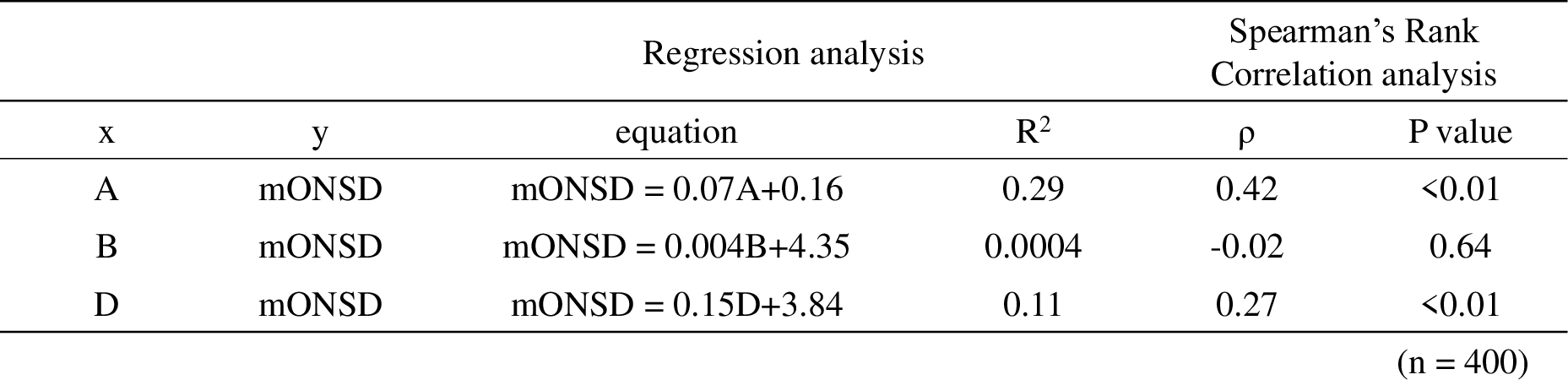

Supplement: S2 Table — (TIF) [file pone.0240845.s002.tif]

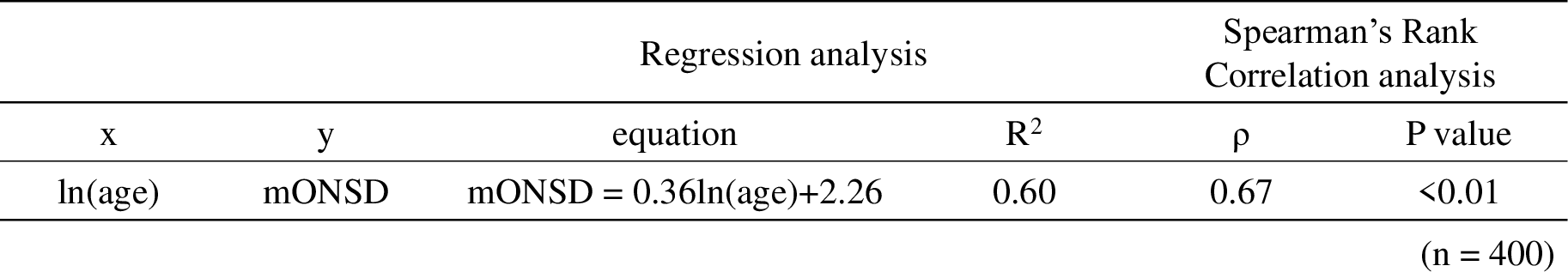

Supplement: S3 Table — (TIF) [file pone.0240845.s003.tif]

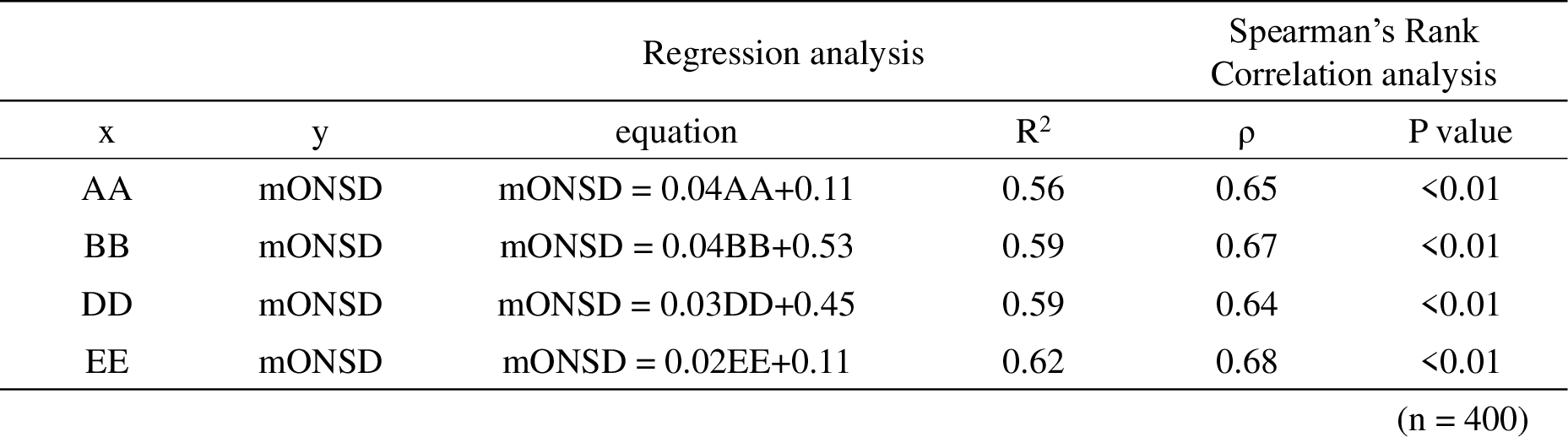

Supplement: S4 Table — (TIF) [file pone.0240845.s004.tif]

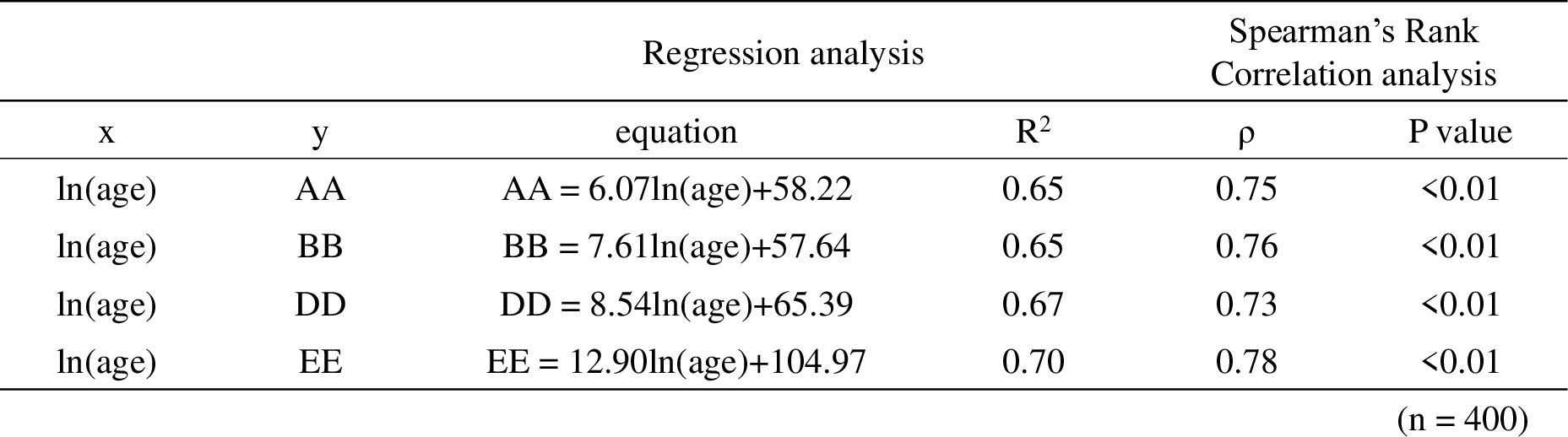

Supplement: S5 Table — (TIF) [file pone.0240845.s005.tif]
